# Links between metabolic syndrome and the microbiome

**DOI:** 10.1093/emph/eoaa007

**Published:** 2020-02-19

**Authors:** Theresa E Gildner

**Affiliations:** Department of Anthropology, Dartmouth College, Hanover, NH, USA

## Abstract

Metabolic syndrome (MetS) is a cluster of harmful conditions which occur together, such as insulin resistance, abdominal obesity, and hypertension. The global prevalence of MetS is growing rapidly, with some estimates suggesting over one billion people worldwide experience increased morality and disease rates linked with this syndrome. One possible factor contributing to MetS risk is changes in microbiome composition. Approximately 100 trillion bacteria and other microbes reside in the human intestinal tract, collectively termed the gut microbiome. Humans and microbes share a long evolutionary history, with many of these microbes influencing human health outcomes. However, environmental conditions have changed dramatically with human technological innovations; many of these changes (e.g., diets high in processed foods and sedentary lifestyles) appear to impact human-microbe relationships. In general, recent changes in diet and activity patterns have been linked to decreased microbiome diversity, elevating inflammation and metabolic disease risk and likely promoting the development of MetS. Targeting patient diet or exercise patterns may therefore help doctors better treat patients suffering from MetS. Still, additional work is needed to determine how the microbiome responds to changes in patient activity and diet patterns across culturally and biologically diverse human populations.

## METABOLIC SYNDROME

Metabolic syndrome (MetS) is a cluster of co-occurring pathological conditions, characterized by insulin resistance, abdominal obesity, hypertension and dyslipidemia [1]. While specific diagnostic criteria differ, in all cases MetS is clearly linked with increased risk of mortality and comorbidities (e.g. type 2 diabetes and cardiovascular disease) [1]. The global prevalence of MetS is growing rapidly, with some estimates suggesting over 1 billion people worldwide are affected by this syndrome [1]. Rising rates of MetS have been largely attributed to high-calorie diets and sedentary lifestyles, yet the physiological mechanisms driving MetS development are not well understood.

## EVOLUTIONARY PERSPECTIVES

One possible factor contributing to MetS risk is changes in microbiome composition. Some 100 trillion bacteria and other microbes reside in the human intestinal tract, collectively termed the gut microbiome [2]. Humans and microbes have co-evolved for over 1 million years, leading to a high degree of interdependency [2]. However, environmental conditions have changed dramatically across human history due to technological advances, with implications for host-microbiome interactions. Specifically, altered living conditions have resulted in an ‘evolutionary mismatch’, defined as differences between ancestral conditions that our species likely experienced across our evolutionary history and the world today. This mismatch includes shifts in diet and activity patterns, which affect energy balance and alter the microbiome, a pattern documented across diverse populations [2, 3]. Diets high in processed foods appear to alter microbiome composition in ways that promote higher fat mass and insulin resistance, perhaps by enhancing energy extraction during digestion [4]. Additionally, evidence suggests that a sedentary lifestyle decreases microbiome diversity, elevating inflammation and metabolic disease risk [5]. On balance, the available evidence suggests that general shifts in diet and physical activity in recent human history may act jointly to alter microbiome composition in ways that enable the onset of MetS. Thus, addressing the core evolutionary mismatch (e.g. through targeting patient diet or exercise patterns) may help doctors better treat MetS.

## FUTURE IMPLICATIONS

In addition to existing pharmaceutical options, interventions designed to alter patient microbiome composition may help prevent or decrease morbidity linked with MetS. For example, the use of probiotics has been shown to change the microbiome in ways that improve metabolic health (e.g. enhancing insulin sensitivity) [4].

Individual-specific exercise regimens may also produce beneficial changes in microbiome diversity, reducing inflammation linked with poor metabolic health [5]. Still, many existing experimental studies have used rodent models, which may not accurately reflect human–microbe co-evolutionary history. Additional work is needed to test how the microbiome responds to modest, attainable changes in diet and physical activity across ecologically and genetically diverse human populations. Medical studies testing these associations will help identify which dietary adjustments and exercise types have the greatest potential to protect patients from MetS.
